# Low-Burden Mobile Monitoring, Intervention, and Real-Time Analysis Using the Wear-IT Framework: Example and Usability Study

**DOI:** 10.2196/16072

**Published:** 2020-06-17

**Authors:** Timothy R Brick, James Mundie, Jonathan Weaver, Robert Fraleigh, Zita Oravecz

**Affiliations:** 1 Department of Human Development and Family Studies Real-Time Science Laboratory The Pennsylvania State University University Park, PA United States; 2 Applied Research Laboratories The Pennsylvania State University University Park, PA United States; 3 Department of Human Development and Family Studies IMPEC Lab The Pennsylvania State University University Park, PA United States

**Keywords:** smartphone apps, ecological momentary assessment, real-time analysis, behavior change

## Abstract

**Background:**

Mobile health (mHealth) methods often rely on active input from participants, for example, in the form of self-report questionnaires delivered via web or smartphone, to measure health and behavioral indicators and deliver interventions in everyday life settings. For short-term studies or interventions, these techniques are deployed intensively, causing nontrivial participant burden. For cases where the goal is long-term maintenance, limited infrastructure exists to balance information needs with participant constraints. Yet, the increasing precision of passive sensors such as wearable physiology monitors, smartphone-based location history, and internet-of-things devices, in combination with statistical feature selection and adaptive interventions, have begun to make such things possible.

**Objective:**

In this paper, we introduced *Wear-IT*, a smartphone app and cloud framework intended to begin addressing current limitations by allowing researchers to leverage commodity electronics and real-time decision making to optimize the amount of useful data collected while minimizing participant burden.

**Methods:**

The *Wear-IT* framework uses real-time decision making to find more optimal tradeoffs between the utility of data collected and the burden placed on participants. *Wear-IT* integrates a variety of consumer-grade sensors and provides adaptive, personalized, and low-burden monitoring and intervention. Proof of concept examples are illustrated using artificial data. The results of qualitative interviews with users are provided.

**Results:**

Participants provided positive feedback about the ease of use of studies conducted using the *Wear-IT* framework. Users expressed positivity about their overall experience with the framework and its utility for balancing burden and excitement about future studies that real-time processing will enable.

**Conclusions:**

The *Wear-IT* framework uses a combination of passive monitoring, real-time processing, and adaptive assessment and intervention to provide a balance between high-quality data collection and low participant burden. The framework presents an opportunity to deploy adaptive assessment and intervention designs that use real-time processing and provides a platform to study and overcome the challenges of long-term mHealth intervention.

## Introduction

### Background

One of the primary strengths of the mobile health (mHealth [1]) movement is its ability to deliver intervention and assessment where and when it is needed and to remain passive, imposing a minimal burden on the participant at other times. For example, ecological momentary interventions [2] and just-in-time adaptive interventions (JITAI [3]) are developed to deliver targeted, adaptive interventions to participants only when needed. This process minimizes the burden on the participants, removing an important barrier to engaging participants in interventions over extended periods and making it possible to efficiently target otherwise difficult long-term goals of maintenance, growth, and rare event prevention. Specifically, mHealth analyses and interventions have targeted maintenance goals such as sustained weight loss [4], treatment for chronic conditions such as generalized anxiety disorder [5], long-term processes such as drug addiction recovery [6], and protective and growth processes to promote thriving [7].

Another related strength of mHealth interventions is the ability to collect data about participants nearly continuously with minimal interruption to their everyday lives [8]. The success of these approaches depends on the ability to predict vulnerable states from these data by modeling the dynamic interactions of static and transient factors. As a wide array of behavioral and physiological data can only be collected via self-reports [9], repeated structured surveys remain the dominant data source [10].

Self-report surveys provide rich and valuable data, but they come at a nontrivial cost in terms of participant burden and dropout. A meta-analysis reported an average adherence level of 78% in children and adolescents [11], but variability is high depending on population, measurement, and engagement approaches. Although some recent examples show response rates over 90% [12], others report dropout of over half the sample [13]. Clearly related to this dropout is the balance between burden and incentive or engagement.

Although simple incentives such as participant payments may suffice for short-term studies, these do not scale to long-term interventions. Alternative approaches to improve engagement have used individualized feedback and visualization [14], badges [15], self-tracking [16], or self-experimentation [17]. Participant burden can be reduced, for example, via passive sensing tools [18]. Methods also exist to balance data cost with burden, for example, by reducing survey size through feature selection [19] or by modeling adherence propensity [20]. Research is still needed to optimize the interaction between participant and technology to maximize engagement and minimize burden while ensuring data quality.

### Objectives

In this paper, we introduced the *Wear-IT* framework [21], a software system designed to study and overcome the challenges of long-term engagement in mHealth settings. Wear-IT uses a combination of passive and active sensors, novel computational architecture, visualization for individual feedback and self-monitoring, and real-time responsiveness to optimize participant and technology interaction. These tools allow the development of mHealth solutions for measurement and intervention to deliver treatment, derive scientific inferences, and promote individual thriving through lifelong engagement in mHealth apps.

## Methods

### Wear-IT

The *Wear-IT* framework [21] is a combination of web interfaces, cloud tools, and smartphone apps designed to carefully balance the data needs of researchers, medical doctors, and clinicians with the burden placed on participants. The overarching goal of the framework is to provide a testbed and deployment tool for the rapid iteration of novel approaches to measurement and intervention to understand human psychology, behavior, and health in real time. Wear-IT is currently deployed in targeted studies; a more general release is planned soon.

### Approach

Wear-IT’s general philosophy has been the distinctive focus on balancing the information received by a data source against the demands on the participant. The initial focus of the Wear-IT project is on increasing engagement and reducing the impact of the tools that are most likely to disrupt and interfere with the everyday lives of participants: structured surveys.

Wear-IT runs on participants’ own smartphones (iOS or Android), avoiding the inconvenience of carrying an additional device and providing benefits for, for example, passive collection of phone usage data. As a result, power, network, drive space, and central processing unit (CPU) must be managed as limited resources and balanced, alongside participant burden, attention, and disengagement, against information gain.

Wear-IT concentrates on the following five primary themes to optimize the interaction between participant and technology: (1) intelligent and responsive scheduling to minimize interruption to daily life, (2) adaptive questionnaire design to reduce response time and effort, (3) custom question types to improve response precision, (4) individualized modeling to adapt to each participant, and (5) individualized visualization and feedback to improve intrinsic motivation and promote self-care and self-experimentation.

The *Wear-IT* framework follows a typical mobile app architecture consisting of four primary components: a smartphone app and sensor monitoring service; a web service for survey management, application programming interface (API) integration, and data collection; a cloud-based storage and computation service for real-time processing; and a visualization generation and delivery engine.

### Smartphone App

Wear-IT has been developed from the ground up to target the tradeoff between resource load and data collected. The smartphone app, therefore, accesses a combination of passive and active sensing tools and is designed to integrate and manage the results of those sensors in real time following a model inspired by concepts from the field of *edge computing* [22].

In our approach, lightweight models run continuously *at the edge of the cloud*—that is, directly on the smartphone or interface device. These models provide efficient real-time responsiveness when network is unavailable but are constrained to have minimal impact on device resources. Heavier computation (eg, individualized model parameter estimation) occurs on the cloud, and the results are used to update the lightweight models.

For example, the app might constantly read streaming data from a wrist-based heart rate sensor and deliver a stressor questionnaire every time some indicator (say, heart rate variability, HRV) crossed a threshold. Later, data would be delivered to a cloud server and combined with historical data to make updated predictions and update or adjust the threshold for that individual. Wear-IT thus delivers real-time, network-independent responsiveness using up-to-date data models without draining resources on the participant’s smartphone.

**Figure 1:**
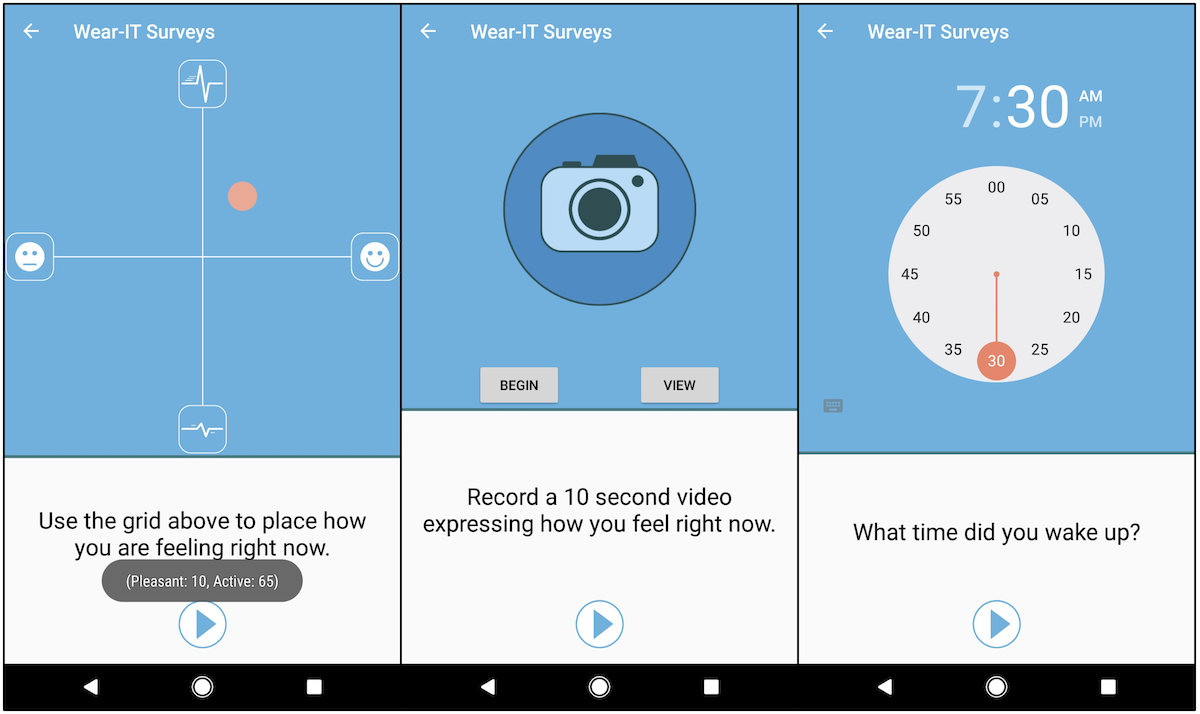
Three example questions from the Wear-IT framework. From left to right: core affect grid, affective video prompt, and time-picker.

As a smartphone-native app, Wear-IT allows researchers and clinicians to develop, test, and deliver questions in formats that are less burdensome or more efficient than traditional approaches. For example, Figure 1 shows examples of three nontraditional survey question types: a two-dimensional *core affect* rating space [23] (similar to the core affect grid in the study by Meers et al [24]), a multimedia recording prompt to collect *affective selfies* or video diaries [25], and an intuitive time picking question.

### Web Server and App Architecture

Wear-IT is designed to allow researchers to quickly create independent studies that customize how surveys and other interventions are delivered to the participants and to control how, when, where, and by what device(s) other data are collected. Researchers define the content of the study’s surveys and provide a set of rules defining the conditions that trigger their delivery to the participant. Study rules also define what types of passive data are collected from the participant’s smartphone and how those data are used. These rules are delivered from a central web server to participant devices in the form of JSON-encoded attributes from a RESTful web API and then interpreted by the Wear-IT smartphone *app* on the wearer’s device. This configuration mimics typical mobile content delivery methods and effectively decouples survey content from survey presentation, allowing for a more robust delivery mechanism. Updates to the Wear-IT app for new versions of mobile operating systems (OSs) or new devices (eg, tablets, wearables that run apps, or internet-of-things [IoT] platforms) require only that an appropriate app be built for that device using that meets the modular requirements of the web service. Thus, Wear-IT is able to adapt to a wide variety of commercially available consumer electronic devices owned by participants. As of the time of writing, Wear-IT has successfully drawn data from devices manufactured by Google, Apple, Garmin, Oura, Fossil, and Empatica; development and testing are still in progress for devices from Fitbit and other third-party devices running Android’s WearOS.

The web server is responsible for distributing survey definitions and other study-dependent information, including managing the rules for how and when the smartphone app delivers surveys and other interventions and the limits of customization for those rules. The app then interprets those rules within the mobile context of individual participants. For example, a study might allocate a time frame for an end-of-the-day survey to be delivered but might allow the participant to customize (within the app) the latest possible survey time to correspond with their usual bedtime. In another study, interventions might be triggered when the participant enters a geographic region of interest but also depend on the participant’s physiological markers, for example, if heart rate is higher than a person-specific threshold. The app is responsible for monitoring the specific conditions, given the hardware configuration available, and delivering the survey. That is, the app might rely on OS-provided geofencing capability to determine when the participant entered the appropriate region or might be required to periodically measure GPS location and compute entry itself. Survey rules also provide specifications about cases where the signal is not available. For example, if a participant opted out of location tracking, they might be removed from the study or might face altered rules for survey delivery that focus on different predictors. The modular balance between server and app and the ability to adapt to different situations makes Wear-IT a versatile platform for data collection and intervention delivery.

The overall architecture of the Wear-IT program is centered around data collection items of different types, called *item types*, which are collected into data collection events. These events include any case where participant data are recorded, including timed surveys, passive collection events, or participant-triggered *one-button* labeling responses. Conceptually, a common scheduling process can be used to organize regular passive collection, random or semirandom, according to triggered events or through some combination of those characteristics. For example, a study might request GPS or Bluetooth-based proximity every 5 min, deliver four surveys per day such that one appears in each of the morning, midday, afternoon, and evening, and trigger additional surveys if a participant reports a craving for alcohol or enters an area indicated as a likely place for a drink. The server architecture converts these conceptual arguments into a common JSON format; the app on each smartphone platform is responsible for implementing the specific requests.

Note that not all data sources are available on every platform. For example, at the time of writing, limitations on beacon libraries reduce the ability of devices running recent versions of iOS to respond to the proximity of other iPhones or to respond to the usage of different app types; by contrast, these services are available on most Android devices. Researchers and clinicians must, therefore, balance the importance of these data streams against the burden of providing participants with an additional smartphone and the data privacy cost of collecting such data.

### Wearable, Passive, and Emplaceable Data Collection Devices

The Wear-IT framework can integrate with a variety of sensors and devices, including wearables, passive smartphone sensors, and emplaced IoT devices such as Bluetooth low energy (BLE) beacons or digital home assistants. At the time of writing, most ongoing studies rely on smartphone-based self-report or self-recording alongside passive tracking of one or more of activity, sleep, heart rate or HRV, stress, smartphone app usage, text messages, location, and proximity to other participants or emplaced objects. It is worth noting, however, that the Wear-IT framework is designed to rapidly incorporate additional measures. For example, several of the Garmin devices we have tested include pulse oximetry. Although this data stream is in theory already available for users of the Wear-IT framework, we have not yet identified a use case for that data stream and so its use within the system remains essentially unapplied and untested.

#### Wearable Physiological Monitors

Wear-IT is capable of integrating with a number of consumer-grade off-the-shelf wearable devices that participants may already own and use daily, such as Fitbit, Garmin, Apple, or Android Wear watches. Although these commercially available wearables may not provide scientific-quality data, they can reduce the cost of data collection and lower participant burden by using socially acceptable devices that participants may already own and use. The individualized modeling approach [12] taken by Wear-IT’s responsive assessment engine is designed to make it possible to *tune* to the differences in predictive power of the device’s data results. Note that consumer devices come with their own drawbacks, including differences in real-time access, privacy, and precision. When validated data collection is necessary, Wear-IT is also able to integrate data from scientific wearables (eg, the Empatica E4 device [26]).

Wear-IT’s approach to integration attempts to meet each device according to its own capabilities and affordances as developed by its manufacturer. As a framework for research, we have designed Wear-IT so that it accesses each tool in a modular fashion, such that common data streams can be harmonized across different devices. In practice, each device has different limitations and a different means of access. For example, the Empatica E4 wristband has a released software development kit for real-time monitoring that permits Wear-IT to directly interpret real-time data about movement, electrodermal activity, skin temperature, and heart rate. By contrast, the Oura ring [27] currently has no published direct access. Although the ring’s smaller form factor and longer battery life are benefits, data must first be uploaded to the Oura servers and then downloaded to Wear-IT servers via OAuth2-authenticated API calls authorized by the participant in the app. As a result, the Oura ring’s data cannot be used to trigger just-in-time interventions. It might, however, be used to adapt an exercise intervention to account for the previous night’s sleep, where the Empatica’s shorter battery life might make such adaptation difficult. Another tradeoff is at the level of control. Some devices running Android’s WearOS, such as many Fossil smartwatches [28], may be able to actively trade off data density with battery life by explicitly controlling sampling frequency using an on-device companion app, which may also provide wrist-based controls. For other devices, such as Garmin watches, Wear-IT relies on the manufacturer’s proprietary smartphone app to collect data from the device and transmit it to a web server; again, the Wear-IT server accesses the data regularly via OAuth2-authenticated API calls. As Wear-IT accesses these devices indirectly, it is not possible to guarantee real-time access to data, although, in general, data are accessible within a short period (eg, a few hours). This later access may be sufficient for cases where, for example, the evening survey is altered based on a person’s daytime location or their peak stress level throughout the day, but may not be sufficient to trigger a mindfulness intervention in response to a stress event. These types of tradeoffs must be managed individually on a per-device basis.

In theory, common data types should yield identical results regardless of the device that makes up the source of the data. In practice, however, there are nontrivial differences in the precision, frequency, and preprocessing pipelines applied to data from different devices. Although the goal of Wear-IT is to seamlessly integrate with an array of devices, at present, data integration is performed on a case-by-case basis. In cases where scientific goals require specific data quality or where integration is difficult for other reasons, devices may need to be provided to participants to ensure consistency or equivalence or limits may need to be applied to the types of data collected based on available sensors.

#### Passive Smartphone Sensors

Owing to their always-on and often-connected nature, smartphones provide a convenient way to gather data that can be used to make automated inferences about the participant’s context with little or no burden and a high degree of accuracy. Modern phone-based data sources such as GPS location, proximity to other participants, and phone usage can be collected through efficient on-phone APIs that have minimal impact on battery life or CPU. Note that some collection, such as raw accelerometry, may come with costs (eg, drive space) that may also need to be balanced.

#### Emplaced Devices

Not all devices that have therapeutic importance are wearable in nature. With the increasing availability of IoT devices, researchers have begun to use motion recording [29] and passive sensors such as BLE beacons [30] to provide vital sensor streams for research and clinical applications. Although most of these sensors must be handled with custom analysis, a few provide clear applications. For example, BLE beacons can provide quick identification of distance to other phones and locations, for example, to identify engagement with a 12-step program or to trigger assessment when a participant enters an area that may constitute a potential relapse trigger. IoT devices such as the Amazon Echo also may provide easy forms of interaction with the participant, whereas tools such as Bluetooth scales or bed-based weight monitors [31] can measure other important health characteristics with minimal participant burden. Again, the specific implementation of these various tools may depend on the specific application and use case. At present, the Wear-IT app framework has limited but increasing ability to interface with emplaced measurement devices.

### Real-Time Adaptation for Responsive Assessment and Intervention

In addition to assembling information for later analysis, the large quantity of passive data collected by the Wear-IT framework is intended to serve as a basis for real-time decision making, for both the framework internally and for clinicians working with the participant. Specifically, Wear-IT aims to leverage low-burden data to determine when and how to collect data that exacts a higher participant burden. Wear-IT’s computing architecture provides passive monitoring with high responsivity and can integrate the results of this monitoring with other active data collection to decide when and how to request new information.

For example, in the beginning of addiction recovery, regular tracking of craving, affect, and sleep are helpful; an intensive assessment strategy may be needed to keep the patient on task. After several years of recovery, however, the patient may only rarely encounter states of high craving and risk, most of which are tied to encounters with, for example, contexts that trigger memories of substance use, such as places of previous high usage [32]. Constant four times a day Ecological Momentary Assessment (EMA) monitoring of craving state at this point would be needlessly burdensome. Instead, Wear-IT is designed to collect passive data to predict risk states and to use it to adapt assessment timing and intensity to match that risk. For example, an additional craving assessment might be delivered when the participant returns to a high-risk area (eg, a bar) and shows signs of physiological stress. The combined active and passive data can then be used to deliver interventions (eg, a mindfulness intervention and an evening recommendation to attend a 12-step meeting). Of particular note, these events and risk states may frequently occur where network access is limited, especially for rural participants.

To promote this type of adaptive responding, Wear-IT provides *contextually driven prompts*: assessments or interventions triggered based on a tree of decision rules. Typically, assessments delivered via contextual prompts are costly in terms of some finite resources such as participant burden, burnout, or test-retest reliability; risks such as disclosure, regulatory concerns, or privacy violations; or technical limitations such as battery, storage, or processing. Rules may combine transformations of contextual factors extracted from the continuous data stream, such as time of day, location, or proximity to other participants, with prior active or passive data and parameters of heavier computations from the cloud server. One simplified but common example might be an EMA data collection (costly in terms of participant burden), which is triggered by a randomized timer. More intricate collections are restricted by decision rules using arbitrary combinations of continuous or periodic data. For example, the app might request information about a location once the participant has visited the same place for a length of time that is determined by a predictive model from the cloud and adjusted regularly as updated estimates become available (eg, following an approach such as MIDDLE [33]). Simple linear models such as logistic regression or even support vector machine–based classification can be implemented in this same decision tree–like framework by simply replacing the single value (eg, heart rate is higher than 100) with a formula-based combination of values. For example, a survey might trigger when the weighted sum *B_1_*×HeartRate+*B_2_*×HRV is higher than a threshold *T*, where *B_1_, B_2_,* and *T* have been determined by fitting a logistic regression or support vector machine. Ongoing research has begun to incorporate more advanced models, such as Bayesian sequential updating models [34], vector autoregressive models [35], and automated feature selection results [19] into this process, although integrating these more intricate tools into Wear-IT is still a work in process.

**Figure 2:**
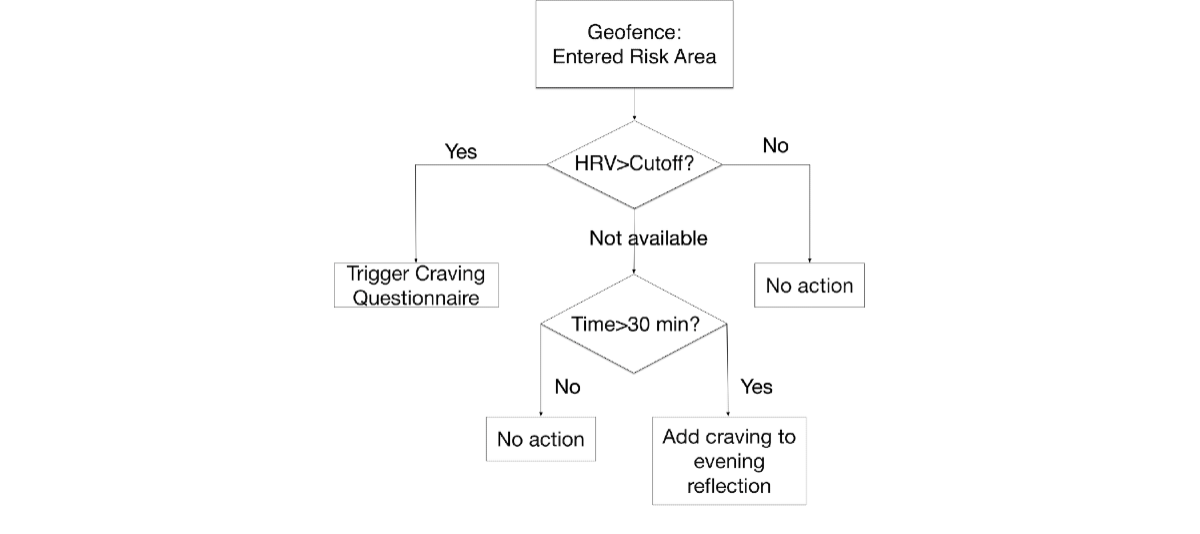
A hypothetical example of a decision tree for triggering different interventions. HRV: heart rate variability.

In brief, these models are kept relatively lightweight by using sequential updating methods whenever possible, by relying on existing OS-level functions when available (eg, for GPS), and by tuning the temporal precision of modeling whenever possible. For example, the rule set provided in Figure 2 uses a preselected set of identified *geofences*—that is, areas of the map that have been precomputed as spaces of interest. Rather than call upon constant GPS tracking (high in battery cost) and performing comparisons in the app itself, Wear-IT can rely on existing OS processes to add geofencing. As the OS manufacturers customize this process to the phone hardware already, we benefit immediately from the result. In this particular case, HRV is also a condition of triggered feedback. As the study more generally records HRV at regular intervals (eg, every time heart rate is sampled), the most recent version can be cached in the app for quick lookup when it is required. If the most recent sample is not sufficiently current, a new sample can be requested via Bluetooth (given availability). This type of active triggering would allow both the wearable and app to remain in a mostly passive state and reduce cost in terms of battery while still remaining responsive and contextually aware.

To provide an example, a responsive intervention (costly in participant burden) in recovery from substance use disorder might monitor proximity to areas where a participant previously reported high cravings (eg, bars or areas they previously used drugs [32]). When in proximity to those areas, the app might begin to monitor HRV from a wearable as a symptom of stress [36], and trigger an intervention if a threshold is crossed. An example of such a decision tree is shown in Figure 2. Notice that the threshold cutoff is not a fixed quantity: this might be changed for each individual and updated weekly from the server to reflect changes in baseline HRV. As another example, automated audio recording [37] (costly in terms of disclosure risk) for a client in addiction recovery might be suppressed when the participant was in a location used for private group therapy.

### Evaluation Approach

We provide two approaches to evaluation of the Wear-IT framework. First, we present a case study of visualization and feedback as a proof of concept and to demonstrate the types of data that Wear-IT can collect and process. Second, we held qualitative interviews with 4 users who are currently deploying studies using the Wear-IT framework. Interviewees included 3 faculty members and 1 graduate student in different departments across the health and behavioral sciences.

## Results

### Visualization and Feedback

**Figure 3:**
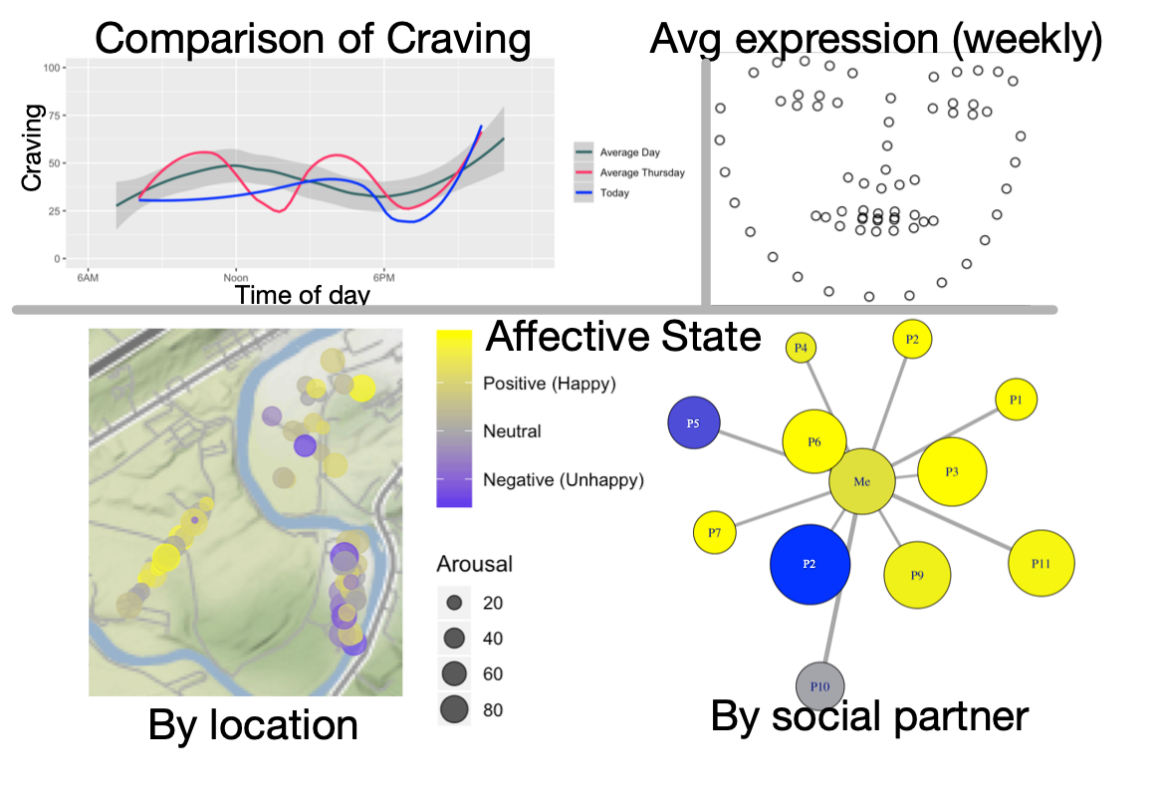
Example visualization of momentary affect and craving from Wear-IT.

Individualized visualization and feedback have been shown to be effective both as a means of engagement [15] and treatment [14]. Although these visualizations must be tailored to the specific study or treatment, a variety of tools are available to display different types of data, such as those displayed in Figure 3. Note that these data are provided for demonstration purposes only and are modified from a true dataset to protect privacy. These do not represent research data and should not be used to drawn generalizable inferences; rather they are intended only to display the types of visualizations in use.

In the top left, a multiline comparison shows a psychological state (here craving, but potentially affect, anxiety, or some other psychological state of interest) across time. The current day (*today*) is compared with other similar days (*average Thursday*) and all days overall (*average day*). Top right shows an averaged facial expression of all emotional expressions (eg, based on selfies) recorded over the course of the week. The bottom two panels show affect (left: both valence and arousal) distributed across location and in response to different interactors over the week (right: closer nodes to the center indicate longer times spent). Together, these tools allow participants to examine the influences on their lives (eg, persons, places, and times). For example, the lower right area of the map indicates an area in which the participant experienced common negative affect; approaching this location might be the right time to deliver an intervention. In the lower left area, persons P2 and P5 on the network plot are persons who induce negative affect; the participant might seek out coping strategies to improve their relationship with these two people or learn to avoid them whenever possible. Similarly, rules might be added to Wear-IT to deliver appropriate JITAIs to assist the participant in applying appropriate coping strategies when they found themselves in such a location or near a given individual.

### Qualitative Interview

In total, 3 faculty members and 2 graduate students agreed to provide feedback in a qualitative interview to relate their experiences using the Wear-IT framework. Note that the resulting data are for product evaluation, and not a part of a formal study. It is not intended to be used for generalizable inference but rather to provide evaluation of the framework in its status as of early 2020. Some researchers additionally reported anonymous quotations from participants engaged in their studies—these data were collected under institutional review board (IRB) approval for those studies using Wear-IT. For clarity, we use the term *users,* meaning researchers using the Wear-IT framework, and *participants* to indicate participants in their studies.

None of the users or participants whose comments are described here were part of the development team or authors on this paper.

Overall, users were highly positive about the ease with which the platform allowed them to pilot, test, update, and deploy new data collection instruments (eg, to adjust survey questions). Participants, too, reported the platform was easy to use. One participant volunteered that they always felt comfortable responding to the prompts provided.

A primary theme across the interviews was the benefit of real-time processing and adaptive responding, including real-time visualizations. A particular point of interest was the ability to create adaptive assessments, although the lack of established best practices for this type of deployment limited ease of use for these more intricate cases. For example, one user on the one hand applauded the ability to deliver adaptive assessments based on proximity triggers but on the other expressed concerns about the complexity required to specify these rule sets (eg, how long must a participant be near a target before they are considered to be *close*).

Although most participants were not informed about the adaptive nature of scheduling, they also highlighted the benefits of adaptive assessment. Users highlighted that their participants were satisfied with the timing of survey assessments, with one participant expressing that they never had to worry about remembering to answer the surveys in question. Although we did not formally evaluate it, we hope that this results in reductions in data missingness in the future.

A second theme that emerged from interviews was excitement about the opportunities that the Wear-IT framework provided them in the design of future studies. Most discussion around this topic focused on the benefits of integrating social and behavioral context into upcoming assessments, the burden reduction inherent in context-responsive assessment or intervention delivery, and the benefits of triggered assessments, especially for cases where substantive questions were related to social interaction or to activities related to real-world locations (eg, recovery communities).

## Discussion

### Principal Findings

The Wear-IT framework provides a new approach to combining passive and active sensing using real-time processing. Although other approaches provide mobile frameworks for survey scheduling and delivery (eg, Ohmage [38,39] and Effortless Assessment of Risk States [40]) and passive data collection, and some tools include the ability to trigger deployment of assessments based on simple decision rules (eg, Sensus [41]), Wear-IT expands on this same primary goal by providing a deeper integration of real-time processing from the outset. The use of arbitrarily complex decision rules to trigger assessment or intervention delivery enables contextual markers more intricate than simple boundary conditions. As real-time processing of contextual information is used by more scientists, we expect that the challenges of specifying these more complex deployments will be simplified by a reduction to common practice. Wear-IT also integrates with a newer wave of wearable devices, such as the Oura ring, to provide new options for data collection that may more easily fit into participants’ lifestyles.

### Participant Privacy

The large amount of data collected by the Wear-IT project raises serious concerns about participant privacy and data security. Wear-IT is designed to be as private as possible while maintaining scientific precision and verifiability. At present, we follow specific guidelines to ensure this type of privacy. First, we protect participant confidentiality by leaving the Wear-IT app in a *white labeled* state whenever possible. The Wear-IT app provides a relatively consistent look and feel across studies in which it is deployed. This allows us to provide surveys and perform data collection without alerting incidental users of the phone to the potential goals of the study. That is, a participant using Wear-IT as a part of a study on addiction recovery will not give away the reason for their participation simply because they use the app itself. Second, we limit data collection to only that data that are required and limit the data available on the phone itself to those elements that are immediately necessary. All scientific data collection must be approved by an IRB to ensure scientific oversight, and any data storage follows the latest security standards and meets all appropriate regulations (eg, applicable requirements of the US Health Insurance Portability and Accountability Act or the European General Data Protection Regulation in Europe). Third, we provide users with as much capability to customize data collection as reasonably possible. This is generally done by requesting permissions for each type of data to be used (eg, GPS and proximity), and by providing users with preferences inside the app that allow them to disable each type of data collection as needed. Of course, limiting GPS data collection may reduce the ability of the app to respond to location-based cues. Hybrid approaches, where they do not conflict with scientific or clinical goals, may also be possible. For example, it is possible to provide users with a map of their GPS-recorded travels for the day and allow them to remove specific locations or routes before their data are uploaded to the server, although this may conflict with real-time responsiveness goals. Again, our goal is to balance participant risks and burden with the scientific and clinical goals of each project. Note that in some cases, it may even be possible to generate responses to data without uploading it.

Finally, no matter how many security precautions are taken, there is some risk that data are disclosed. Although we take as many precautions as possible to reduce this risk, it must still be recognized and balanced against gains. As a result, we consider the risk of disclosure to be another specific type of burden that must be balanced with the others. That is, just as there is burden on the user incurred by taking the time to respond to a survey, there is burden in the risk of disclosure incurred storing the result on the server. We follow the same principals in balancing this cost as we do the other types of cost (eg, technical costs such as battery life and psychological costs such as questionnaire burden). As with the others, we use a combination of context-sensitive adaptation, specific instructions, and real-time processing to limit the identifiability of data whenever possible.

As an example of this last approach, consider a study in which participants record daily *video diaries*, which are then processed to understand a participant’s affective state. This type of data collection involves a variety of privacy risks. First, facial expression video of the participant themselves contains sensitive information about their identity, and there is some risk of the participant disclosing information that is more sensitive than they wish to share or information that might be overheard by others nearby. Second, the background behind the person’s face includes potentially identifying location information. Third, it is possible that other people in the area might also be recorded, possibly without their express knowledge or permission. As with other cases, it is important to balance the value of the data against the risk and cost to the participant. A variety of options exist to manage this type of risk. For example, if we know the specific information we want to collect from the video, for example, affective state variables, all three concerns could be mitigated by applying facial expression tracking to the video on the phone and sending only a processed stream of coded affective signals (eg, happy and sad) or a version cropped only to the participant’s face, in lieu of raw video. This might carry additional burden in the form of processor time and battery life in exchange for greater privacy protection. In cases where this is not possible, video recording might be triggered via geofencing so that it is requested only when the user was in their home or via beacon proximity when they were in, for example, a private area of a recovery community. In this case, the generalizability of the experiences recorded might be limited in exchange for greater privacy protection. If the clinical or scientific needs still do not allow this type of adaptation, instructions might simply be provided, asking the participant to check their surroundings and seek out a space that limits those risks in which they can answer the questions. In any of these cases, participants should be provided with the opportunity to review the video after they record it and to delete it before upload if desired.

The rise of mHealth methodology has provided a large number of new tools and processes for scientific data collection and clinical intervention. The combination of passive and active sensing approaches available to these tools makes them perfect choices to balance the informational needs of scientific inquiry and adaptive intervention with the challenges related to participant engagement and burden to target the long-term requirements of treating chronic and long timescale processes. However, mHealth tools are rarely designed to collect data or deliver interventions for these long-term projects. In this paper, we presented the Wear-IT framework, an app framework designed to leverage real-time processing of active and passive measurement to optimally balance resources, engagement, and data quality for clinical intervention and scientific inquiry.

## Abbreviations

API: application programming interface
BLE: Bluetooth low energy
CPU: central processing unit
EMA: Ecological Momentary Assessment
HRV: heart rate variability
IoT: internet-of-things
IRB: institutional review board
JITAI: just-in-time adaptive interventions
mHealth: mobile health
OS: operating system
